# Effects of Adjuvant Exercise and Nutrition Therapy on Muscle Fibre Biomechanics in Gastrointestinal Cancer Patients

**DOI:** 10.3390/cancers16081608

**Published:** 2024-04-22

**Authors:** Michael Haug, Raphaela Schwappacher, Charlotte Pollmann, Paul Ritter, Mena Michael, Hans Joachim Hermann, Robert Grützmann, Anke Mittelstädt, Markus Friedrich Neurath, Yurdagül Zopf, Oliver Friedrich

**Affiliations:** 1 Institute of Medical Biotechnology (MBT), Department of Chemical and Biological Engineering, Friedrich-Alexander-University Erlangen-Nürnberg, Paul-Gordan-Str. 3, 91052 Erlangen, Germany; charlotte.pollmann@fau.de (C.P.); paul.p.ritter@fau.de (P.R.); mena.michael@fau.de (M.M.); oliver.friedrich@fau.de (O.F.); 2 Erlangen Graduate School in Advanced Optical Technologies (SAOT), Friedrich-Alexander-University Erlangen-Nürnberg, Paul-Gordan-Str. 6, 91052 Erlangen, Germany; 3 Hector-Centre for Nutrition, Exercise and Sports, Medical Department 1, Friedrich-Alexander-University Erlangen-Nürnberg, Ulmenweg 18, 91054 Erlangen, Germany; raphaela.schwappacher@uk-erlangen.de (R.S.); hans.hermann@uk-erlangen.de (H.J.H.); yurdaguel.zopf@uk-erlangen.de (Y.Z.); 4 Medical Department 1, Friedrich-Alexander-University Erlangen-Nürnberg, Ulmenweg 18, 91054 Erlangen, Germany; markus.neurath@uk-erlangen.de; 5 Department of General and Visceral Surgery, Friedrich-Alexander-University Erlangen-Nürnberg, Krankenhausstr. 12, 91054 Erlangen, Germany; robert.gruetzmann@uk-erlangen.de (R.G.); anke.mittelstaedt@uk-erlangen.de (A.M.); 6 School of Biomedical Sciences, University of New South Wales, Wallace Wurth Building, 18 High St, Sydney, NSW 2052, Australia

**Keywords:** cancer cachexia, single fibres, skeletal muscle, multi-modal therapy, resistance training, protein-rich diet

## Abstract

**Simple Summary:**

Our study examined how muscle function is affected in cancer patients, particularly those at risk of developing muscle wasting (cachexia). We tested a combination of mild exercise and a protein-rich diet as a potential preventive measure. By analysing muscle fibres from patients undergoing different treatments, we found that cancer cachexia leads to muscle weakness and stiffness. However, our results show that the combined therapy helps maintain muscle flexibility and function. This suggests that such an intervention could improve the quality of life for cancer patients by preserving muscle health.

**Abstract:**

Patients with aggressive cancer, e.g., gastrointestinal cancer, are prone (≥50% chance) to developing cancer cachexia (CC). Little is known about the effects of CC on the biomechanical function of muscle. A promising prevention strategy was found in the form of a multi-modal therapy combining mild resistance exercise (e.g., whole-body electro-myostimulation, WB-EMS) and a protein-rich diet. In a previous study of ours, this was effective in counteracting the loss of muscle mass, yet a systematic and comprehensive assessment of active and passive single muscle fibre functions was so far absent. This pilot study investigated the biomechanical function of single muscle fibres (*rectus abdominis*) from the biopsies of conventionally treated (pre-)cachectic cancer ((pre-)CC) patients (m = 9), those receiving the multi-modal therapy comprising WB-EMS training and protein-rich nutrition (m = 3), and a control group (m = 5). Our findings not only align with previous findings showing the absolute force loss in CC that is accelerated by atrophy but also speak in favour of a different, potentially energy- and Ca^2+^-homeostasis-related effect that compromises muscle contraction (F ~0.9 mN vs. F ~0.6 mN in control patients). However, myofibrillar Ca^2+^ sensitivity and the quality of contraction were unaltered (pCa50: 5.6–5.8). Single fibres from the (pre-)CC patients receiving WB-EMS training and protein supplementation were significantly more compliant (*p* < 0.001 at ≥130% of resting length L_0_). Those fibres displayed a similar softness to the ones from the control patients (axial compliance ~15 m/N at ≥130% L_0_), while single fibres from the patients with (developing) cachexia were significantly stiffer (axial compliance ~7 m/N, *p* < 0.001 at ≥130% L_0_). Adjuvant multi-modal therapy (WB-EMS training and nutritional support) contributes to maintaining the axial compliance of single fibres and potentially improves the quality of life for patients at risk of developing CC.

## 1. Introduction

Roughly 10–20% of patients affected by cancer cachexia (CC) die from it [1]. Patients with gastrointestinal cancers are at high risk of developing CC [2] (≥50% of patients with pancreatic cancer, gastric cancer, or colorectal cancer [2]). CC is characterised by a progressive loss of muscle mass [3]. The severity of CC is reflected by increased mortality, higher surgical risk, and a diminished response to conventional cancer treatment, e.g., chemotherapy [4,5,6]. A person’s current muscle mass is the result of a balance between protein synthesis and breakdown [3]. In CC, this equilibrium is shifted towards increased muscle breakdown, dysfunction, and impaired muscle repair [7], which are caused by multi-organ energy imbalance due to insufficient nutrient supply and chronic inflammation [8]. This under-/malnourished state is a combination of reduced food intake and tumour-induced altered metabolism and is characterised by the constant release of pro-inflammatory cytokines [9]. Conventional treatment strategies (radio-/chemotherapy) further accelerate the progression of CC by impeding physical activity and nutrient absorption [10]. Counteracting these degenerative processes and contractile dysfunctions to enhance anabolic processes and maintain skeletal muscle mass and function in cancer patients is crucial [11]. The catabolic burden of the disease impairs protein requirements; thus, a high daily protein intake of 1.0–2.0 g/kg body weight is recommended [12]. The benefits of physical exercise on skeletal muscle are well known, and patients with cancer are motivated to exercise. Clinical studies with cancer patients suggest that exercise improves body composition, physical function, and inflammation [13]. Unfortunately, whole-body physical activity often proves difficult for advanced-stage cancer patients, which is the major reason for the scarcity of comprehensive studies [9]. Whole-body electro-myostimulation (WB-EMS) is an innovative, efficient, and less strenuous form of resistance exercise. If employed prior to engaging in physical activity, it helps patients with limited mobility to maintain or increase skeletal muscle mass and promote anti-inflammatory pathways and has been confirmed as a resourceful adjuvant therapy in, e.g., osteopenia and sarcopenia [14].

**Figure 1:**
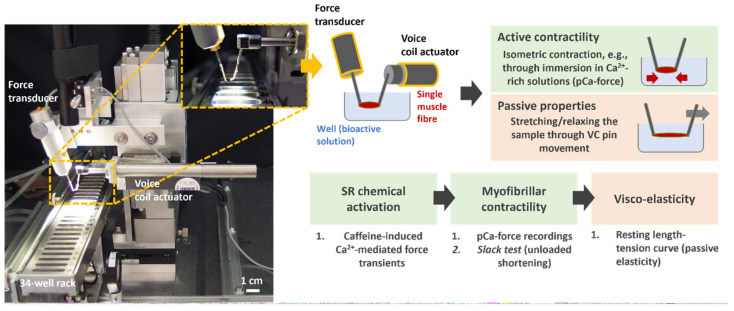
Muscle performance assessment at the single-cell level is carried out with the *MyoRobot* biomechatronics assessment platform by analysing human *rectus abdominis* single muscle fibres. The *MyoRobot* consists of a force transducer and a voice coil actuator (highlighted in a dashed rectangle) between which a single fibre is mounted (silk-threaded micro-knots tied to the fibre and around each pin). The pictograph to the right displays how the fibre is immersed in bioactive solutions, for which the rack consists of 34 individual wells. Motorised control allows the device to automatically assess the sample’s active and passive biomechanical properties. In this study, we controlled the assessment of excitation–contraction coupling functionality, myofibrillar contractility, and passive elastic properties in an automated fashion to elucidate the biomechanical performance and kinetics at the single-cell level.

Recently, we demonstrated the positive effects of such a multi-modal treatment on increasing muscle mass in advanced-stage cancer patients [13]. We showed that a 12-week therapy with WB-EMS and high-protein nutrition stabilises or enhances the participants’ skeletal muscle mass and physical functioning [13]. However, deciphering the CC-induced alterations in skeletal muscle cytoarchitecture and systematic effects on single fibre biomechanics represents a missing piece in understanding the whole impact of CC on muscle dysfunction, atrophy, and weakness. For the first time, we present a pilot study conducted with non-tumour control patients vs. patients with gastrointestinal tumours who either received or not received WB-EMS and nutritional intervention. Single muscle fibres from biopsies were subjected to biomechanical measurements (sarcoplasmic reticulum (SR) Ca^2+^ release activation, myofibrillar Ca^2+^ sensitivity, contractility, and (visco-)elasticity) using our *MyoRobot* (Figure 1) [15]. The few available studies suggest aggravated force production [16] and impaired Ca^2+^ sensitivity of the contractile apparatus [17]. However, systematic effects are so far elusive since no single study has ever assessed both active and passive contractile properties from the same single-fibre preparation from (pre-)cachectic patients. Our *MyoRobot* technology is well equipped to close this gap [18] and, in this study, it was applied to investigate the systematic effects of (developing) CC and a multi-modal exercise and nutrition therapy approach on active and passive muscle biomechanics at the single-cell level.

## 2. Materials and Methods

### 2.1. Patients

**Figure 2:**
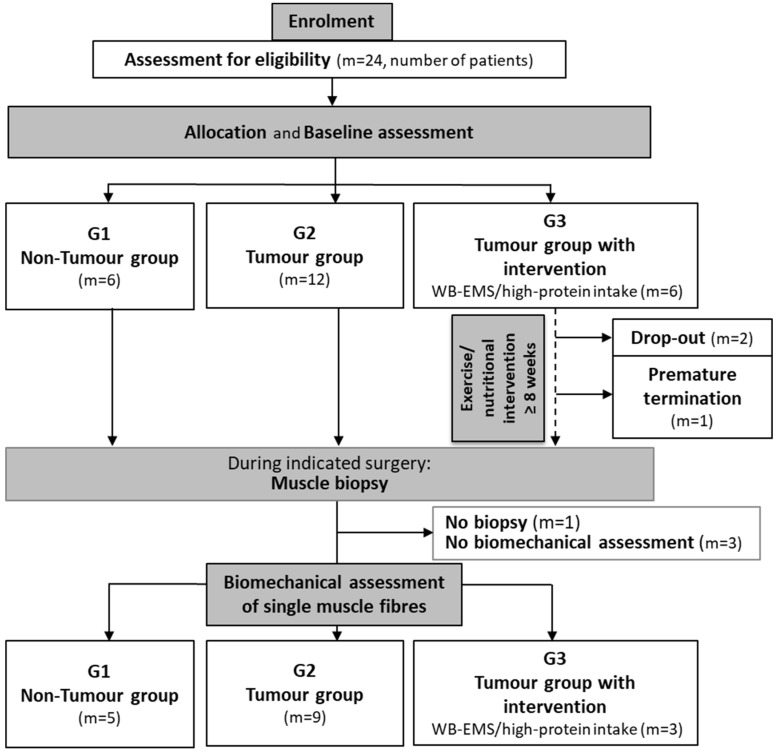
Study design and decision tree. The flowchart outlines the allocation of patients into study groups. G1 comprises non-tumour patients; G2 comprises tumour patients receiving conventional treatment; and G3 comprises tumour patients receiving additional WB-EMS training and a high-protein diet. During an indicated surgery, a biopsy was taken for biomechanical assessment. Patient numbers and, therefore, biopsy numbers, are given as ‘m=’.

This study included patients (≥18 years) with solid pancreatic, colorectal, oesophageal, or gastric tumours (UICC stage I–IV) under oncological treatment and tumour-free patients (Figure 2). Written consent was obtained. Recruitment occurred between June 2017 and November 2021. All included patients required an indicated surgery proximal to the *M. rectus abdominis*. The following patients were excluded:

- Being in other nutrition or exercise intervention studies;
- Study-independent exercise ≥ once a week;
- Ingestion of anabolic or dietary supplements;
- Occurrence of heavy cardiovascular events;
- Epilepsy;
- Severe neurological disorders;
- Skin lesions around the electrodes;
- Conductive materials or electronical implants in the body;
- Pregnancy;
- Chronic diseases (e.g., HIV or Hepatitis C/D/E).

### 2.2. Study Design and Multi-Modal Therapy

At study entry, blood samples were collected. Blood analysis included markers for the following:

- Inflammation (C-reactive protein, CRP, normal value < 5 mg/L);
- Nutrition (albumin, 35–55 g/L);
- Renal function (creatinine, 0.51–1.17 mg/dL);
- Haematological parameters (leucocytes, 4.4–11.3 × 10^3^/μL; thrombocytes, 150–300 × 10^3^/μL; erythrocytes, 4.1–6.0 × 10^6^/μL; haematocrit, 35–48%; and haemoglobin, 11.5–18.0 g/dL).

Unintentional weight loss within the last six months was recorded as evidence of (pre-)CC (>2% and ≤5%) and CC (>5% weight loss) [19]. After the baseline assessment, non-tumour patients were allocated to study group 1 (G1). Tumour patients with imminent surgery were assigned to study group 2 (G2), and tumour patients with an indication for surgery within +8 weeks were allocated to study group 3 (G3), allowing some time to deploy WB-EMS training and a high-protein diet. Within this period, the patients underwent resistance exercise training in the form of WB-EMS with high-protein dietary support (>1.0 g/kg body weight) [13]. WB-EMS training was performed under professional supervision twice a week for 20 min. The electrodes of the WB-EMS equipment (vest, upper arm cuffs, upper thigh cuffs, and hip belt (miha bodytec GmbH, Gersthofen, Germany)) administered bipolar impulses (85 Hz, pulse width of 350 μs, with 6 s impulse phase followed by 4 s rest) with a low current intensity to stimulate the muscles of the upper arms, upper back, latissimus dorsi, chest, abdomen, lower back, buttocks, and thighs [9,13]. The current intensity was individually adapted to each muscle region and was set to trigger an apparent muscle contraction.

### 2.3. Biopsy and Sample Processing

A 1 × 1 × 1 cm sample of the *rectus abdominis* muscle was dissected at the beginning of the surgery. The specimen was transferred to the biomechanics laboratory in cold (8 °C) Ringer’s solution. The sample was pinned onto a PDMS-coated Petri dish (SYLGARD 184^®^ Silicone Elastomer Kit, Dow, Texas City, TX, USA). Ringer’s solution was exchanged for a calcium-free, high-potassium solution (HKS) to permanently depolarise the cell membrane and render the specimen inexcitable and relaxed. The sample was allowed to rest at 8 °C for 30 min, bubbled with air, before single muscle fibre segments were dissected. Both ends of a fibre segment (~2 mm) were tied to a silk micro-knot and mounted between the force transducer and voice coil actuator on the *MyoRobot*. The sample was lowered into a relaxing ‘idle’ solution (1% *w*/*v* high-relaxing solution in a low-relaxing solution, see ‘Chemical Solutions’) [18].

### 2.4. Physiological Solutions

The specific composition of the physiological solutions is given in the supplementary information in Haug et al. [20]. The solutions were thawed from stocks on the day of the experiments. These solutions were freshly supplemented with creatine kinase (CK, Sigma-Aldrich/Merck KGaA, Darmstadt, Germany) to ~300 U/mL or ~3 U/well for ATP re-synthesis and 0.1 M sodium azide to prevent mitochondrial Ca^2+^ uptake. Each single muscle fibre was exposed to a saponin solution (0.1% (*w*/*v*) saponin in HR solution) for 20 s to permeabilize the sarcolemma. The solutions used were as follows:

- **High-activating solution (HA)**: Ca^2+^-saturated environment to chemically induce maximum force generation ([Ca^2+^]_free_~12 μM).
- **High-relaxing solution (HR)**: strong Ca^2+^-chelating (EGTA) environment to buffer excess Ca^2+^.
- **Low-relaxing solution (LR)**: the high Ca^2+^-chelating EGTA was exchanged for the low-affinity HDTA prior to any subsequent solution exposure.
- **Loading solution (LS)**: the previously emptied SR was re-loaded for a defined time (consisting of HA and HR titrated to [Ca^2+^]_free_~300 nM).
- **Release solution (RS)**: a total of 30 mM caffeine was added to LR, which triggered SR Ca^2+^ release. The force transient was proportional to the releasable SR Ca^2+^ content.
- **pCa solutions**: the Ca^2+^ sensitivity of the contractile apparatus was assessed for defined pCa values (mixture of HA and HR; calculated using React (Geoffrey Lee, University of Glasgow).

### 2.5. Active and Passive Biomechanics Recordings with the MyoRobot

**Figure 3:**
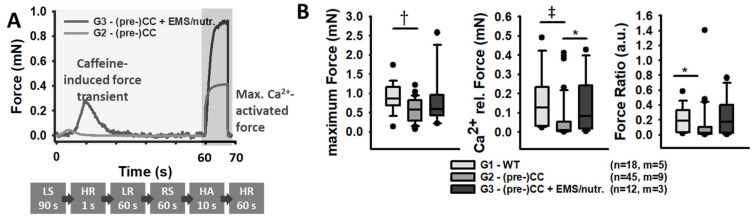
SR Ca^2+^-release-induced force is significantly diminished in (pre-)CC patients, while single fibres from WB-EMS-treated patients with protein-rich nutrition perform similarly to fibres from non-tumour patients. (**A**) Representative recording in a single fibre from a (pre-)CC patient with (G3) or without (G2) receiving the multi-modal treatment. The sequence of the recording protocol is shown below (see Methods for solution abbreviations). (**B**) The maximum Ca^2+^-activated force, the Ca^2+^-release force, and the ratio of both. Level of significance is indicated as follows: *: *p* < 0.05; †: *p* < 0.01; and ‡: *p* < 0.001, while n = ‘number of recorded single fibres’ and m = ‘number of individual patients/biopsies’. Error bars: SEM.

**Figure 4:**
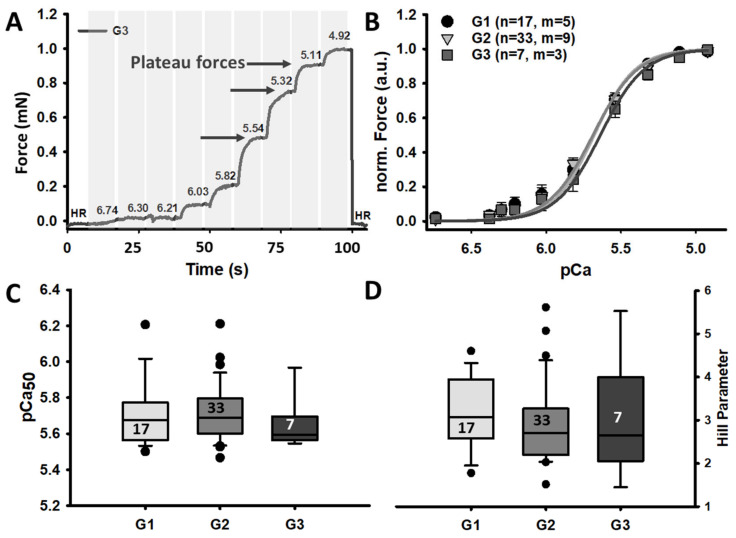
Unaltered sensitivity of the contractile apparatus to externally applied Ca^2+^ in (pre-)CC patients with (G3) or without (G2) adjuvant multi-modal treatment. (**A**) Representative recording of a single fibre from a (pre-)cachectic patient receiving adjuvant multi-modal treatment (G3). (**B**) The average peak forces for each executed pCa step of all study groups, along with their average reconstructed fit curve. (**C**,**D**) The pCa_50_ value and the Hill parameter, respectively. n = ‘number of recorded single fibres’ and m = ‘number of individual patients/biopsies’. Error bars: SEM.

**Figure 5:**
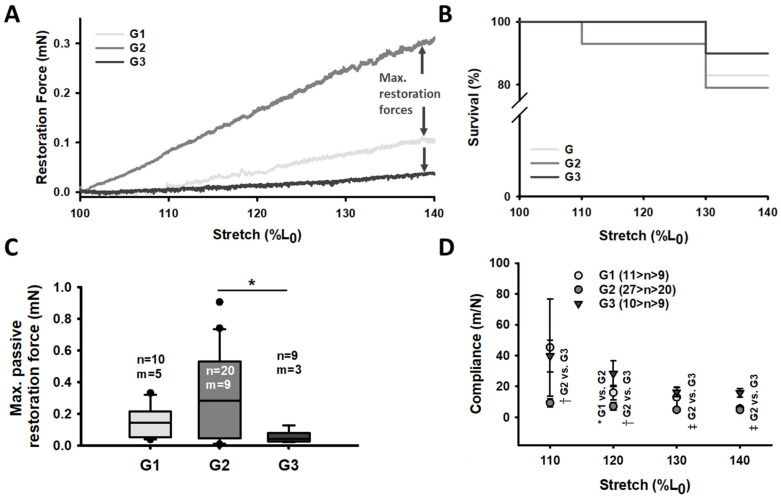
Significantly increased single muscle fibre axial stiffness and passive restoration force in (pre-)cachectic patients not receiving multi-modal treatment. (**A**) Passive axial elasticity was investigated by stretching a single fibre under relaxing conditions to 140% of its resting length within 30 min. (**B**) The Kaplan–Meier survival plot of the procedure. (**C**) The maximum passive restoration forces at 140% L_0_ (so-called ‘resting length–tension curve’). (**D**) The passive axial compliance. Level of significance is indicated as follows: *: *p* < 0.05; †: *p* < 0.01; and ‡: *p* < 0.001, while n = ‘number of recorded single fibres’ and m = ‘number of individual patients/biopsies’. Error bars: SEM.

The *MyoRobot* is a robotised system that specialises in muscle performance diagnostics [18]. Its 34-well rack allows the mounted specimen to be exposed to bioactive solutions after membrane permeabilization. By combining the force transducer (TR-5S, Myotronic, Heidelberg, Germany) and voice coil actuator (CAL 12, SMAC Inc., Munich, Germany, Figure 1) technology, the *MyoRobot* can assess most active and passive biomechanical properties of muscle (from the whole organ to the single cell) at room temperature. The sequential experiments were carried out as follows:

- **Caffeine-induced, Ca^2+^-mediated force transients:** The fibre was exposed to HR to wash off saponin and excess Ca^2+^ buffer before exposure to LR. It was then submerged in LS for 90 s to load the SR. The caffeine-induced force transient was triggered in caffeine-rich RS for 60 s. Eventually, the maximum force was triggered in Ca^2+^-saturated HA solution for 10 s (see Figure 3A,B).
- **Ca^2+^ sensitivity of the contractile apparatus:** the specimen was consecutively exposed to solutions of increasing Ca^2+^ concentrations (decreasing pCa (−log_10_[Ca^2+^])) for a duration of 10 s each (see Figure 4A).
- **Passive axial stiffness/compliance:** the muscle fibre was stretched at 0.44 μm/s in the LR solution to 140% L_0_ (see Figure 5A).

### 2.6. Data Analysis and Statistics

The patients’ characteristics were analysed using GraphPad Prism 9.5.1 (GraphPad Software, San Diego, CA, USA; RRID: SCR_002798). For statistical analysis of normally distributed data, one-way ANOVA with Tukey’s multiple comparison test was used. For non-parametric analysis, the Kruskal–Wallis test with Dunn’s multiple comparison test was applied. All *MyoRobot* data were analysed in RStudio (RStudio Inc., rstudio.com, Boston, Massachusetts, USA [21], RRID:SCR_000432). Data presentation and follow-up statistical analyses were performed with SigmaPlot version 14 (Systat Software Inc., sigmaplot.co.uk, San Jose, CA, USA, RRID:SCR_003210), abiding by the same distinctions made with Prism. Level of significance was indicated as follows: *: *p* < 0.05; **: *p* < 0.01; and ***: *p* < 0.001, while n = ‘number of recorded single fibres’ and m = ‘number of individual biopsies’. Detailed data analysis for each biomechanics recording was as follows, while a moving average smoothing was applied to any force trace:

- Caffeine-induced, SR Ca^2+^-release force transients: Force data were read-in and corrected for their baseline (force in HR solution). The plateau force was determined by the 99% quantile. The force ratio of the Ca^2+^-release peak force to maximum Ca^2+^-saturated force was calculated (see Figure 3A).
- Ca^2+^ sensitivity of the contractile apparatus: The 99% quantile was used to determine the plateau forces at each pCa step (Figure 4B). The forces were normalised to the maximum force and plotted against the pCa values. A four-parameter Hill equation ($y=y_{0}+\frac{a*10^{-bx}}{c^{b}+10^{-bx}}$) was fitted to the data, where a = 1 and y_0_ = 0. The Hill coefficient (b) and the pCa_50_ value (−log_10_([c]) were utilised to reconstruct a mean fit to the average data points (Figure 4B).
- Passive axial stiffness/compliance: At 140% L_0_, the maximum passive restoration force was determined. Linear fits were applied to every section of 10% stretch. The slope represents the passive axial stiffness. Its inverse is the fibre’s axial compliance (see Figure 5A).

## 3. Results

### 3.1. Study Design and Patient Data

Due to surgical reasons, one participant (G1 with six participants) could not donate a muscle biopsy (Figure 2). G1 patients underwent surgeries for peptic stenosis (1), hernia (1), pancreatitis (1), or diverticulitis (2). G2 comprised twelve tumour patients with imminent cancer-related surgery; muscle biopsies were obtained from nine of these patients (gastric/oesophageal (6), pancreatic (1), and rectal carcinoma (2)). Half of these patients might have suffered from CC (unintentional weight loss confirmed). Muscle biopsies from three participants in G2 could not be analysed. From G3 (six participants), two participants dropped out (lack of interest), and one patient prematurely left the study (deteriorating physical condition). The remaining patients (gastric/oesophageal (2) and rectal carcinoma (1)) received WB-EMS training twice a week and a high-protein diet (>1 g protein/kg body weight) daily. A third of these patients seemed to suffer from CC (unintentional weight loss prior to the study). The pre-surgical intervention period was ≥8 weeks; the mean intervention time was 9.0 ± 4.0 weeks, with 14.3 ± 8.8 WB-EMS sessions. The patients’ characteristics are presented in Table 1.

### 3.2. Maximum Ca^2+^-Saturated Force and Caffeine-induced, SR Ca^2+^-Release Force Are Compromised in (Pre-)CC Patients but Ameliorated by Adjuvant Multi-Modal Therapy

Exposure to Ca^2+^-rich solution revealed a significantly compromised force in (pre-)CC patients not receiving the adjuvant exercise and nutrition therapy (G2, Figure 3A) in comparison to the single fibres of tumour-free patients (G1) and those receiving the adjuvant exercise and nutrition therapy (G3). In G3, the adjuvant treatment recovered the otherwise notable force loss and restored the maximum force generation to levels comparable to those of non-tumour control patients (G1). Yet, due to the comparatively small sample size of individuals in G3 (n = 12 fibres; m = 3 patients), this trend could not be substantiated by the statistical analysis. The observed trend of reduced force in G2 vs. G3 was more pronounced when investigating SR functionality in terms of SR Ca^2+^ release (immersion in 30 mM caffeine solution). The mechanism of chemico-mechanical coupling appeared to be significantly compromised in the single fibres from (pre-)CC patients (Figure 3B). The single fibres from G1 and G3 patients still produced a caffeine-induced, Ca^2+^-mediated force transient of 20% of their maximum myofibrillar force. This force transient was almost absent in the single fibres from the conventionally treated (pre-)CC patients (G2).

### 3.3. Ca^2+^ Sensitivity of Rectus Abdominis Single Fibres Seems Unaffected in Patients with or without Adjuvant Multi-Modal Therapy

To investigate whether the diminished force in (pre-)CC patients originated from altered myofibrillar Ca^2+^ sensitivity, the pCa–force relationship was investigated. Much like the closely matched average data points for each distinct pCa step, the mean reconstructed fit curves are also similar (Figure 4B). The pCa_50_ values of all treatment groups were centred between 5.6 and 5.8, revealing no significant alteration in the contractile apparatus’ graded response to different Ca^2+^ levels (Figure 4C). This robustness of myofibrillar Ca^2+^ sensitivity is also reflected by the dynamic range of the graded force response (Hill parameter). The median Hill parameters of all treatment groups ranged from 2.5 to 3.0 without a significant difference (Figure 4D). It seems that a (pre-)cachectic condition neither impacts on the Ca^2+^ sensitivity nor the dynamic response range to Ca^2+^ of single muscle fibres from the human *rectus abdominis* muscle.

### 3.4. Passive Axial Single Muscle Fibre Stiffness Is Increased in (Pre-)CC Patients without Multi-Modal Adjuvant Treatment

In addition to the diminished active force generation, we observed a marked fibre stiffening in the single fibres from (pre-)CC patients not receiving the adjuvant exercise and nutrition therapy (G2). This increased stiffening and larger resistance towards stretch impacted the overall survival of single fibres in this protocol (Figure 5B). (Pre-)cachectic single fibres (G2) were the least robust (75% survival) and were outperformed by G1 control fibres (83%) and G3 single fibres (90%). The maximum passive restoration force of fibres at 140% resting length (L_0_, Figure 5C) was similarly low for G1 and G3 (~0.1–0.2 mN). The fibres from (pre-)CC patients (G2) displayed a four-times, and therefore, significantly increased restoration forces. Similar results were found for the fibres’ axial compliance/flexibility (Figure 5D): G1 and G3 fibres displayed values within the same range and compliance declined with stretch, while in the fibres from G2, compliance values decreased and did not decrease notably with stretch.

## 4. Discussion

Defining the onset of CC is a vivid debate. Some definitions were postulated by Bozetti [22] (habitual weight loss of ≥10%), Schwarz [23] (lower endurance capacity), Illman [24] (elevated levels of inflammatory cytokines), and Fearon [19] (weight loss ≥ 5%, or ≥2% if BMI < 20 kg/m^2^). Yet, the development and the condition of CC often precede diagnosis. Therefore, we favour an inclusive terminology for (pre-)cachexia ((pre-)CC). Understanding skeletal muscle catabolism and anabolism is key in treating (pre-)CC [25] since prolonged inflammatory processes upregulate genes that promote apoptosis and protein degradation [25]. To account for this, the ESPEN guidelines on nutrition in cancer patients suggest a high protein intake of 1–1.5 g/kg BW daily [26]. Yet, the ideal quantity and optimal amino acid composition remains to be determined. Likewise, physical activity provides some protection against cancer, e.g., via myokines with anti-tumour activity [9]. Therefore, it is likely that the inflammatory profile preceding CC impacts on skeletal muscle cytoarchitecture and biomechanical function. This was systematically assessed in the present study in human *rectus abdominis* single muscle fibres using biomechanics recordings with the *MyoRobot*.

### 4.1. Compromised SR Ca^2+^-Release-Induced Force and Maximum Force Are Ameliorated in (Pre-)CC Patients Receiving Adjuvant Multi-Modal Therapy

In CC, the literature reports a force decline between 30 and 50% that varies amongst different muscles [17]. The observed significantly reduced (~40%) maximum force in (pre-)cachectic patients matches the findings of comparable studies. Although the WB-EMS training and nutritional support seemed to decelerate or prevent the progressive force loss in (pre-)CC patients, this trend could not yet be substantiated by the statistical analysis. Nevertheless, single fibres from patients receiving the combined exercise and nutrition therapy produced active forces like those of tumour-free control patients (~1 mN, Taskin et al. (2014) [27]; ~4 mN in control *rectus abdominis* fibre bundles of 2–3 single fibres). Several studies have attributed a loss of skeletal muscle myosin to this force loss [16,17,27], which seems to be a major catalyst for myofibrillar degradation and atrophy in critically ill and CC patients. Thus, a diminished myosin-to-actin ratio would result in fewer cross-bridges and a compromised force [17], as observed in our experiments. A reduced actin–myosin content would also imply a thinner fibre diameter (atrophy) and, therefore, absolute force loss. Unfortunately, the current study did not include optical assessment of single fibre atrophy, and we cannot attribute the absolute force loss in G2 to atrophy alone. Other mechanisms might be related to the chemico-mechanical coupling (from SR Ca^2+^ release to motor protein activation). Here, altered Ca^2+^ homeostasis within the SR or changes in myofibrillar Ca^2+^ sensitivity might influence force in the cachectic and pre-cachectic states [7,27]. Therefore, we investigated SR Ca^2+^-release-induced force in caffeine-induced force transients. We observed a significant difference between fibres from (pre-)CC (G2) patients and control (G1) or WB-EMS/nutrition-supplemented (pre-)CC patients (G3). These findings suggest impairments either in the SR’s Ca^2+^ storing or release functionality, a loss of the myofibrils’ Ca^2+^ sensitivity, or both. Intriguingly, endoplasmic or SR stress-related malfunctions, like disturbed Ca^2+^ homeostasis or altered muscle protein translation in patients with cancer or myositis, have been described in the literature [7,28,29]. Here, altered gene and protein expressions of, e.g., calsequestrin 1 and an overexpression of SR Ca^2+^-ATPase (SERCA1) have been associated with muscle weakness and dysfunction. Since calsequestrin 1 is the most abundant Ca^2+^ buffer within the SR, and SERCA1 pumps are not only responsible for Ca^2+^ re-uptake [28], but also a major source of leakage [30], a potentially altered gene expression might contribute to the observed reduction in Ca^2+^-release-mediated force by compromising the intra-luminal Ca^2+^ concentration and homeostasis [7,28]. Such a myoplasmic Ca^2+^ overload within myofibres was recently detected and proposed as a driving force for muscle damage [28], which may represent an indicator for an altered Ca^2+^-storing or Ca^2+^-release capability. An impaired SR Ca^2+^-release capability may contribute to the far-reaching effects seen in CC, such as decreased mitochondrial efficiency, which normally provides a control mechanism for undesired local SR Ca^2+^ release [29]. The observed significantly reduced SR Ca^2+^-release force in (pre-)CC patients (G2), which was ameliorated through the WB-EMS training and nutritional support (G3), speaks in favour of such a theory and supports the idea of an increased myoplasmic Ca^2+^ deposition in (pre-)cachectic muscle tissue. Likewise, it is known that crosstalk between SR and mitochondria impacts on SR Ca^2+^ release/reuptake and ATP utilisation during muscle contraction [29,31]. In that regard, impaired SR Ca^2+^ release would compromise mitochondrial ATP production, leading to energy deficiency during excitation–contraction coupling [31] and reduced active force generation. Nevertheless, this explanation remains inconclusive so far and requires further experiments for verification.

### 4.2. Unaltered Ca^2+^ Sensitivity in (Pre-)CC Patients Suggests Unaltered Quality of Contractility

To exclude the possibility that the loss in Ca^2+^-release-induced force was not the result of altered myofibrillar Ca^2+^ sensitivity, we examined the pCa–force relationship. In contrast to previous studies reporting either Ca^2+^ desensitisation [17] or Ca^2+^ sensitisation [27], our findings suggest an unaltered pCa_50_ and, thus, sensitivity. This suggests that an altered Ca^2+^ homeostasis of the SR [7,28] rather than a shift of myofibrillar Ca^2+^ sensitivity contributes to a diminished active force in (pre-)CC. This is controversially discussed in the literature, particularly regarding the potential contribution of in-/decreased Ca^2+^ sensitivity to active force generation. Myosin isoform shifting seems to present a reasonable explanation for potentially altered Ca^2+^ sensitivity. Less oxidative type II fibres could positively influence Ca^2+^ sensitivity [16,17] and induce a stronger and faster force production [32]. Such a shift would align with the idea of compensating for atrophy-induced force loss [32], yet most studies on CC patients reported unaltered type 1 or type 2 fibre distributions compared to non-cachectic cancer/control patients [33,34]. Although we did not perform single muscle fibre type assessment, our data favour the hypothesis of an unaltered myosin isoform distribution and no major compensation of force through increased Ca^2+^ sensitivity.

### 4.3. Increased Passive Stiffness in Single Muscle Fibres from (Pre-)CC Patients Receiving No Multi-Modal Adjuvant Intervention

Since protein degradation and prolonged inflammation are associated with fibrosis in CC, a change in resistance towards passive axial stretch was expected in the study cohort G2. Increased fibrosis and elevated collagen levels in the skeletal muscle of pancreatic CC patients are a major driving force for structural remodelling [28]. Remodelling is expressed by, e.g., a dilated SR or disintegrated Z-disc and M-line proteins [25]. By investigating these effects on passive axial compliance, we found a significantly increased restoration force and reduced axial compliance in (pre-)cachectic patients (G2) vs. those receiving the adjuvant WB-EMS/nutrition treatment (G3). Restoration force was also notably increased in G2 compared to G1, but this remains to be confirmed as being significant. Intriguingly, single fibres from patients receiving the WB-EMS and high-protein supplementation displayed an even further reduced passive restoration force and higher compliance in comparison to tumour-free control patients. This indicates that WB-EMS training plus high-protein supplementation ameliorates tumour- and inflammation-induced tissue stiffening and exerts a positive effect on the axial compliance of muscle and single muscle fibres, as proposed in sports science [35]. Thus, a major finding of our study is that multi-modal adjuvant WB-EMS/nutrition therapy softens single fibres in (pre-)cachectic patients.

Although fibrosis is a consequence of impaired muscle regeneration, it seems unlikely to be the sole cause for single fibre stiffening because the recordings here were conducted at the single-fibre level, which was free from extracellular matrix-connected neighbouring fibres. Yet, structural remodelling due to muscle damage is evident in CC [28,36]. A loss or a disruption of sarcomeres and their passive elastic proteins, such as titin, α-actinin, and nebulin [25], or myofibrillar remodelling in hampered regeneration cycles as in muscular dystrophy [21], could provide an explanation. Such disrupted force transmission might also contribute to compromised active force generation. Nevertheless, real-time assessment of SL during functional recordings was not yet possible with the *MyoRobot*, at the time of this study, as opposed to more recent studies using an improved *MyoRobot 2.0* [37]. A follow-up study addressing this will be conducted in due course. In general, our findings align well with those of Judge et al. in 2018 [28] by complementing their findings from the functional side. In that setting, our observed (pre-)cachectic muscle stiffness could potentially arise from a (pre-)cachectic impaired muscle repair mechanism that would, in the long term and on the whole-organ level, act as a mediator for fibrosis and increase stretch resistance.

There are limitations to this study, with the first and foremost limitation being the small sample size. Biopsies from patients are scarce, especially in connection with *ex vivo* single muscle cell assessment. Few studies have investigated muscle performance at this level and have mostly centred on obtaining proof of feasibility for a method that is limited to 4–5 individuals [7]. As such, a translation of their results requires more extensive investigations, while also considering the analysis separately, e.g., based on gender. Further, due to inclusive design and recruitment of patients at risk of developing CC and those already diagnosed with CC, a precise conclusion for each respective sub-group cannot yet be made. As a consequence of the sample size, our study does not allow us to draw a distinction between patients receiving neo/adjuvant chemo/radiotherapy, which may influence the outcomes of WB-EMS and nutritional support. In addition, a large-scale study would require a quality-of-life assessment via questionnaires to connect the data to the subjective physical well-being of each individual. Lastly, quantifying how atrophy influences the diminished force observed in this study remains to be conducted, which is a feature we can now employ with our new *MyoRobot 2.0.*

## 5. Conclusions

Decomposing the interwoven role of skeletal muscle in CC presents a current challenge towards a comprehensive understanding and development of potential treatment and prevention strategies. We provide functional insights into the effects of a multi-modal therapy approach combining exercise and dietary supplementation through biomechanics recordings. The most striking effect was the substantial increase in axial stiffness in single fibres from (pre-)CC patients, while there was preserved axial compliance in single fibres from non-tumour control patients and (pre-)CC patients receiving WB-EMS and a high-protein diet. Myofibrillar degradation and structural remodelling, as seen in CC patient [25,28], is likely a key determinant of the increased single fibre axial stiffness seen in (pre-)cachectic patients and potentially accelerates disease progression. By showing that exercise and nutrition therapy can ameliorate some crucial expressions of (pre-)cachexia on the biomechanics of single muscle fibres, we could verify the importance of this adjuvant therapy and provide support to institutionalise such pro-active measures for patients at risk of developing CC.

## Figures and Tables

**Table 1:** Patients’ characteristics. Values are presented as mean (SD). Where appropriate, statistical analysis between the three study groups was performed using one-way ANOVA with Tukey’s multiple comparison test (a) or Kruskal–Wallis test with Dunn’s multiple comparison test (b). For pre- and post-intervention comparison within the exercise group G3, paired *t*-test (c) or Wilcoxon signed-rank test (d) was applied. The superscript e depicts that the neoadjuvant chemo- and/or radiotherapy had just begun at the time of recruitment into the study. *p*-values in bold mark statistically significant differences.

	Study Groups				*p*-Value			
Characteristics	G1 Non-Tumour (n = 5)	G2 Tumour (n = 9)	G3 Tumour with WB-EMS/Nutrition (n = 3)					
			Pre	Post	G1 vs. G2	G1 vs. G3	G2 vs. G3	G3 Pre vs. Post
Sex					-	-	-	-
Male, n (%)	3 (60%)	7 (77.8%)	1 (33.3%)	-				
Female, n (%)	2 (40%)	2 (22.2%)	2 (66.6%)	-				
Age (y)	61.2 ± 10.0	62.3 ± 8.0	62.7 ± 14.1	-	0.976 a	0.976 a	0.999 a	-
Tumour, stage (UICC)					-	-	-	-
I, n (%)	-	2 (22.2%)	1 (33.3%)	-				
II, n (%)	-	1 (11.1%)	1 (33.3%)	-				
III, n (%)	-	4 (44.4%)	1 (33.3%)	-				
IV, n (%)	-	2 (22.2%)	0 (0%)	-				
Oncological therapy				-	-	-	-	
Chemotherapy, n (%)	-	2 (22.2%)	2 (66.7%) ^e^	-				
Chemo- and radiotherapy, n (%)	-	5 (55.6%)	1 (33.3%) ^e^	-				
No therapy, n (%)	-	2 (22.2%)	0 (0%) ^e^	-				
Body parameters								
Body weight (kg)	75.5 ± 15.6	73.4 ± 7.7 (n = 8)	83.8 ± 17.1	85.8 ± 18.1	0.956 a	0.516 a	0.343 a	0.072 c
Weight loss in last 6 months (%)	0 ± 0	10.1 ± 9.1 (n = 7)	3.3 ± 5.7	-	**0.049** b	>0.999 b	0.575 b	-
Body mass index (kg/m^2^)	26.0 ± 2.6	24.6 ± 1.9 (n = 8)	27.9 ± 4.4	28 ± 4.6	0.641 a	0.453 a	0.127 a	0.070 c
Blood parameters								
Albumin (g/L)	42.2 ± 5.4	30.8 ± 6.0 (n = 7)	39.8 ± 2.7	30.4 ± 2.5	**0.011** a	**0.013** a	0.81 a	0.08 c
C-reactive protein (mg/L)	5.6 ± 7.1	42.4 ± 63.2 (n = 8)	4.8 ± 4.8	59.3 ± 92.5	0.534 a	0.448 a	0.907 a	0.421 c
Creatinine (mg/dL)	0.9 ± 0.2	0.8 ± 0.1	0.8 ± 0.2	0.74 ± 0.2	0.464 a	0.546 a	0.986 a	0.274 c
Haematocrit (%)	41.6 ± 3.5	35.4 ± 5.3	39.7 ± 1.9	35.3 ± 6.5	0.102 a	0.199 a	0.99 a	0.312 c
Haemoglobin (g/dL)	13.9 ± 1.3	11.8 ± 1.7	13.0 ± 0.6	11.6 ± 2.2	0.091 a	0.162 a	0.973 a	0.37 c
Leucocytes (×10^3^/µL)	8.2 ± 2.0	6.8 ± 1.5	8.7 ± 4.2	8.9 ± 4.4	0.701 b	>0.999 b	>0.999 b	>0.999 d
Erythrocytes (×10^6^/µL)	4.9 ± 0.4	4.0 ± 0.7	4.7 ± 0.2	3.9 ± 0.8	0.078 b	0.146 b	>0.999 b	0.25 d
Thrombocytes (×10^3^/µL)	341.8 ± 88.0	288.8 ± 100.5	187.3 ± 54.0	183.3 ± 49.6	0.943 b	0.063 b	0.276 b	0.75 d

## Data Availability

The data that support the findings of this study are available from the corresponding author, M.H.
